# Association Between Dietary Variety and Masticatory Behaviors Measured Using Wearable Device Among Community-Dwelling Older Adults in Japan: A Multilevel Meal-by-Meal Analysis

**DOI:** 10.3390/nu17040695

**Published:** 2025-02-15

**Authors:** Kana Eguchi, Maki Shirobe, Masanori Iwasaki, Keiko Motokawa, Tatsunosuke Gomi, Lena Kalantar, Misato Hayakawa, Ayako Edahiro, Hiroyuki Sasai, Shuichi Awata, Hirohiko Hirano

**Affiliations:** 1 Research Team for Promoting Independence and Mental Health, Tokyo Metropolitan Institute for Geriatrics and Gerontology, Tokyo 173-0015, Japan; eguchi1013@icloud.com (K.E.); mshirobe@tmig.or.jp (M.S.); iwasaki@den.hokudai.ac.jp (M.I.); gomi@tmig.or.jp (T.G.); ba7le7@gmail.com (L.K.); mh24@tmig.or.jp (M.H.); aedahiro@tmig.or.jp (A.E.); sasai@tmig.or.jp (H.S.); h-hiro@gd5.so-net.ne.jp (H.H.); 2 Department of Preventive Dentistry, Faculty of Dental Medicine and Graduate, School of Dental Medicine, Hokkaido University, Sapporo 060-8586, Japan; 3 Integrated Research Initiative for Living Well with Dementia, Tokyo Metropolitan Institute for Geriatrics and Gerontology, Tokyo 173-0015, Japan; awata@tmig.or.jp; 4 Dentistry and Oral Surgery, Tokyo Metropolitan Geriatric Hospital, Tokyo 173-0015, Japan

**Keywords:** dietary variety, masticatory behaviors, wearable device, older adults

## Abstract

**Background:** Consuming a variety of foods is believed to promote thorough chewing; however, it remains unclear whether individuals who consume various foods actually chew them thoroughly. Therefore, this study aimed to examine the association between dietary variety and masticatory behaviors, measured using wearable devices, among community-dwelling older adults. **Methods:** Participants were from the Itabashi Longitudinal Study of Aging, meeting the eligibility criteria, including the ability to exchange messages via smartphone or computer. Masticatory behaviors (number of chews, chewing duration, and speed) and meal photo data were objectively measured using an ear-worn bite sensor and its application for two or three meals per day for at least three days at home. The “modified Dietary Variety Score (m-DVS)” (range 0–10, with higher values indicating greater dietary variety) was calculated by registered dietitians. Generalized linear mixed models assessed the association between m-DVS as the exposure variable and masticatory behaviors as the outcome variable. Covariates included sociodemographic status, health behavior, health status, oral health, and oral function. **Results:** Five hundred and eighty-seven mealtime data entries from 63 participants were included in the analysis. The m-DVS was significantly positively associated with the number of chews (cycles, unstandardized regression coefficient = 116.5, 95% confidence interval [CI] = 85.2 to 147.8) and chewing duration (min, unstandardized regression coefficient = 1.7, 95% CI = 1.3 to 2.2). **Conclusions:** Consuming more varied food groups was associated with more chews and longer chewing duration among community-dwelling older adults, potentially promoting thorough chewing.

## 1. Introduction

Frailty refers to a state of increased vulnerability to stress caused by decreased physiological reserve in old age, making individuals more susceptible to outcomes such as impaired life functioning, the need for long-term care, and death. The frailty cycle proposed by Fried et al. shows that declines in physical function, including sarcopenia, along with fatigue and low basal metabolic rate, create a vicious cycle that leads to progressive frailty [1]. A greater variety of food intake is associated with a lower likelihood of experiencing physical frailty [2], and consuming a varied diet is important for healthy longevity [3]. Individuals with a more varied food intake are also more likely to have nutritionally balanced meals and a higher intake of proteins and micronutrients [4].

Frailty is also independently associated with masticatory performance, regardless of nutritional intake [5]. The mouth is the entry point for food, where mastication (chewing) occurs. Chewing cuts, grinds, and mixes food with saliva to form a food bolus that is suitable for swallowing [6]. Thorough chewing (chewing well) promotes saliva secretion [7], which helps form a food bolus favorable for digestion. In addition, thorough chewing offers several benefits: it prolongs meal duration and reduces eating rate [8,9], which can aid in weight management. Chewing also improves masticatory function by increasing the mass of the masticatory muscles [10]. In addition, previous studies [11,12,13,14] suggest that chewing positively influences cerebral blood flow, alleviates stress, and regulates cognitive function. Overall, chewing is thought to be a favorable masticatory behavior. The Ministry of Agriculture, Forestry, and Fisheries (MAFF) of Japan has set the goal of increasing the number of citizens who chew thoroughly [15].

Some studies have shown no association between masticatory function and nutritional intake [16], whereas others have demonstrated an association [17]. Since masticatory behaviors are influenced by the food taken into the mouth, consuming a variety of foods promotes thorough chewing. The MAFF recommendations also mention the consumption of a variety of foods [15]. Regarding masticatory behaviors, food hardness and texture are associated with the number of chews when the same food is consumed [18]. However, the association between a well-balanced diet (i.e., consuming a variety of food groups) and masticatory behaviors, as recommended in daily diet guidelines, remains unexplored. In the past, objective evaluation of masticatory behaviors has not been possible. However, recent advances in sensory and communication technologies have enabled the development of wearable devices that permit monitoring of masticatory behaviors in unsupervised settings, such as daily meals. With this capability, we can now examine the association between the nutritional balance of the meal and masticatory behaviors in daily life. By clarifying this association, valuable insights can be provided regarding the promotion of proper chewing and the improvement of overall health. Therefore, we conducted this study to examine the association between the variety of food intake and masticatory behaviors measured using wearable devices on a meal-by-meal basis among community-dwelling older adults. We hypothesized that consuming a greater variety of foods is associated with chewing more thoroughly (i.e., more chews during meals).

## 2. Materials and Methods

### 2.1. Study Design and Participants

This study is a cross-sectional study based on an ancillary survey of participants in the Itabashi Longitudinal Study on Aging (Itabashi-LSA) [19,20]. Itabashi-LSA is an ongoing cohort study of 70- to 85-year-old residents of the Itabashi ward, located in the northwest area of 23 special wards in Tokyo, Japan. The purpose of the Itabashi-LSA is to generate evidence that will contribute to the realization of a healthy and long-lived society. The detailed protocol for Itabashi-LSA has been explained elsewhere [19]. Those who completed an on-site survey at Itabashi-LSA 2023 were also asked to participate in the current study by mail. Exclusion criteria are as follows: having a history of stroke, having cognitive impairment defined as a Mini-Mental State Examination score ≤ 27, use of a pacemaker, inability to exchange messages via a smartphone or computer, participation in another ancillary survey in the Itabashi-LSA and having incomplete missing study enrollment data. The purpose of the study was fully explained in writing and orally to those who wished to participate. Written informed consent was obtained from all participants. This study was approved by the Ethics Committee of the Tokyo Metropolitan Institute for Geriatrics and Gerontology (approval number: R22-058).

### 2.2. Survey Items

#### 2.2.1. Masticatory Behaviors Measured Using a Wearable Device

**Figure 1:**
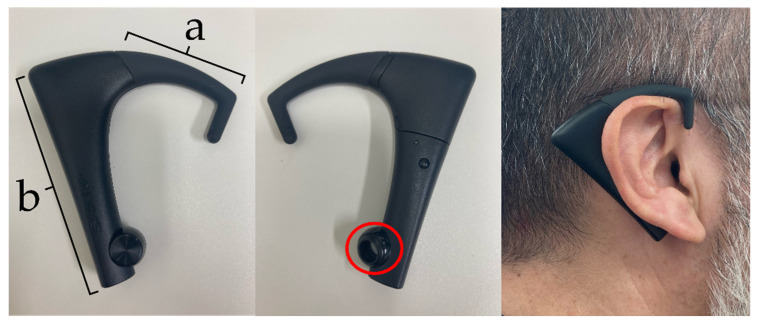
Ear-worn bite sensor (bitescan^®^, Sharp Corporation, Sakai, Japan). (**a**) Variable ear hook; (**b**) body. The device is designed to be worn on the right side of the head. The lower part contains a sensor (circled in red).

Masticatory behaviors at mealtime were measured using an ear-worn bite sensor (bitescan^®^ [BS], Sharp Corporation, Sakai, Japan) [21]. The BS consists of (a) the variable ear hook and (b) the body. This device is designed to be worn on the right side of the head (Figure 1). The lower part contains a sensor. The ear hook comes in three sizes (small, medium, and large), and we selected the size that best fits the participant’s auricle so that the built-in infrared distance sensor and accelerometer could detect morphological changes in the skin surface on the posterior side of the pinna at a mastication frequency of 20 Hz. Masticatory behavior data were recorded using a dedicated application on a smartphone (SH-RM15; Sharp Corporation, Osaka, Japan) connected to the device via Bluetooth.

The BS and smartphone were given to the participants, and the operation method was explained to them so that they could measure the data at home. The BS was calibrated and worn on the right ear. Participants then took measurements at least three days during their meals at home. Meals were limited to their usual content and did not have to be consecutive for more than three days. A previous study has reported that observing a screen displaying real-time BS measurements increases the number of chews [22]. Therefore, we did not instruct the participants on how to chew, but instructed them not to look at the BS measurements while eating.

Specific items of masticatory behaviors, including the number of chews, chewing duration, and chewing speed, were defined as follows:

Number of chews: the total number of chews throughout an entire meal.

Chewing duration: the total time spent chewing before finishing the meal.

Chewing speed: the number of chews divided by the chewing duration.

The validity of the BS for measuring the number of chews has been previously verified through comparison with a mandibular motion-measuring device [20], which measures mandibular movements and counts the number of chews. The accuracy of the BS was 101.6 ± 13.6%, when the test foods of gum, gummy jelly, and rice balls were eaten.

#### 2.2.2. Dietary Variety

Dietary variety was determined by trained registered dietitians based on photographs taken during each meal. Participants took photos of their meals from directly above to capture the entire meal. If a single shot could not capture the entire meal, multiple photos were taken. This study assessed dietary variety from photographs to calculate nutritional intake [23]. Ten food groups—meat, fish and shellfish, eggs, soybean products, milk and dairy products, green and yellow vegetables, seaweed, fruits, potatoes, and oils and fats [24]—were scored as 1 point (present in the meal) or 0 points (absent in the meal). Adding the individual scores generated the “modified Dietary Variety Score (m-DVS)” (range: 0–10 points). A previous study that examined the association between a 3-day dietary record and dietary variety score (DVS) reported a positive correlation between DVS and the frequency of meals that included a main meal, main dish, and side dishes [4]. Additionally, a checklist assessing the number of food groups consumed per meal is disseminated in the community [25,26].

Ten food groups were selected based on the DVS, one of the most widely used indices for assessing dietary variety in the older adults in Japan [24]. This index was evaluated based on the total frequency of intake of food groups consumed almost every day of the week. Therefore, the DVS provides a comprehensive assessment of dietary variety in the daily diet. The DVS has already been utilized in the community to prevent frailty and is attracting attention as a self-checking method for older adults [25,26].

Although dairy products are not included in the DVS, they were included alongside milk in the m-DVS assessment in this study for the following reasons. Previous studies conducted with community-dwelling older adults have indicated that the creation and dissemination of checklists that include dairy products can increase dietary variety [25,26]. When adapting to the community context, dairy products were included in the evaluation in the present study, considering that individuals who are lactose intolerant cannot consume milk. Therefore, one point was added if milk or dairy products, or both, were included.

#### 2.2.3. Covariate Data Collection

Sex, age, educational status (years of schooling), number of household members, and perceived financial situation were assessed using self-administered questionnaires. The number of household members was divided into three categories: “one person”, “two people”, and “three or more people”. For the perceived financial situation, respondents were asked “How is your household’s current living situation?” They were asked to choose from the following options: “Very comfortable”, “Comfortable”, “Average”, “Difficult”, or “Very difficult”.

Weight and height were measured, with the participants wearing light clothing and no shoes. The body mass index (BMI) was computed by dividing the weight in kilograms by the squared height in meters (kg/m^2^).

Comorbidity status was determined through medical interviews conducted by trained nurses, and the Charlson Comorbidity Index (CCI) [27] was calculated. The CCI is an evaluation index that scores comorbidities, with higher scores indicating a poorer prognosis (range, 0–24).

Trained dentists and dental hygienists assessed the oral health and function, including the number of natural teeth, masticatory performance, and occlusal force. The number of natural teeth refers to the number of remaining teeth, excluding the residual roots (range, 0–32). Masticatory performance was evaluated with a gummy jelly (Test Gummy Jelly for Evaluating Masticatory Performance, UHA Mikakuto Co., Ltd., Osaka, Japan) [28]. Without limiting the chewing side, the gummy jelly was freely chewed for 30 strokes and then discharged into gauze, and the score was calculated by comparing the gummy jelly after chewing with visual materials (range 0–9, with lower scores indicating a lower masticatory performance.). The occlusal force was measured three times using dedicated equipment (Oramo-bf; Sumitomo Riko Co., Ltd., Aichi, Japan), and the maximum value was used for analysis [29]. Measurements for denture wearers were obtained with the dentures in place.

### 2.3. Statistical Analysis

Dietary variety and masticatory behaviors per meal were evaluated using meal image data and BS. Since meals were the unit of our analyses, a multilevel model was used to control for correlations between meals within the same participant. First, the characteristics of the study participants and their meals are described. Second, the association between dietary variety and masticatory behaviors was examined using a generalized linear mixed model (GLMM) with a normal family and identity link [30,31], which covers multilevel models. The outcome variables included masticatory behaviors (number of chews, chewing duration, and chewing speed). The exposure variable was m-DVS. Covariates included sex, age, years of education, number of household members, perceived financial situation, BMI, CCI, number of natural teeth, masticatory performance, and occlusal force. Covariates were determined from oral health and function, which could be confounded by factors identified from previous studies [17,32], in addition to basic information such as sex, age, and BMI. In univariate analyses, masticatory behaviors were set as the primary outcome, m-DVS as a fixed effect, and individual participants as a random effect. Only random intercepts were included as random effect blocks. Multivariable models were constructed as follows. Model 1 was adjusted for sex and age. Model 2 was further adjusted for years of education, number of household members, perceived financial situation, BMI, and CCI. Models 3 to 5 were further adjusted for oral health and function. As the numbers of natural teeth, masticatory performance, and occlusal force were highly correlated, these variables were included separately in the model. Subgroup analysis by sex was also conducted using the GLMM.

Statistical analyses were performed using IBM SPSS Statistics for Windows (version 29.0; IBM Corp., Armonk, NY, USA). Statistical significance was set at *p* < 0.05.

## 3. Results

### 3.1. Participant Characteristics

**Figure 2:**
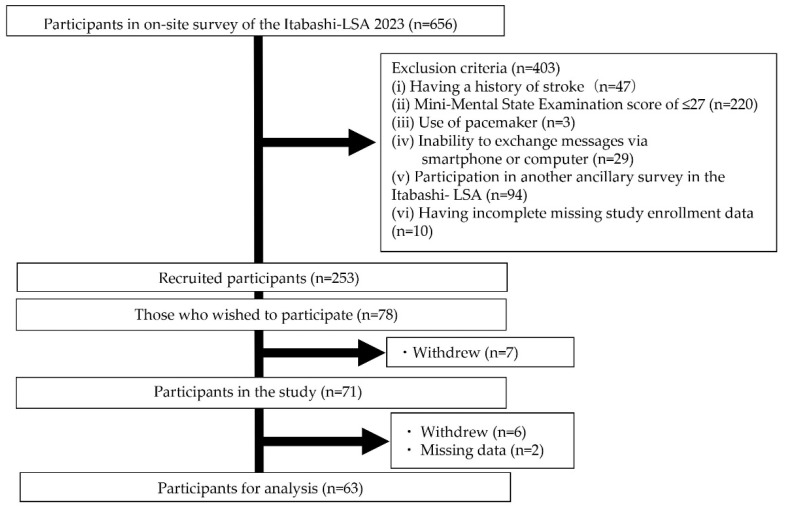
Participant recruitment.

The participant recruitment process is illustrated in Figure 2. Of the 656 participants who underwent an on-site survey using the Itabashi-LSA 2023, 403 met the exclusion criteria. In total, 253 individuals were mailed to participate in the study. Of them, 78 responded positively to the invitation. After receiving a detailed explanation of the study, seven individuals declined their consent to participate. Seventy-one participants participated in this study. Excluding 6 participants who withdrew during the study and 2 who had missing data, 63 were included in the analysis (36 men and 27 women; mean age: 74.4 [standard deviation {SD} = 3.1] years) (Table 1).

### 3.2. Characteristics of Mealtime Data

The participants submitted 591 mealtime data entries (masticatory behaviors measured using the BS and photos of meals). Of these, 587 mealtime data were included in the analysis after excluding four mealtime data entries with missing meal photo data. Mean (SD) values for masticatory behaviors and food groups are presented in Table 2. Among the food groups, “Oil and fats” was the most frequently included (n = 457, 77.9%), followed by “Green and yellow vegetables” (447, 76.1%) and “Meat” (328, 55.9%) (Table 2).

### 3.3. Association Between Dietary Variety and Masticatory Behaviors

The GLMM results are listed in Table 3. In the univariable model, m-DVS was significantly positively associated with the number of chews (unstandardized regression coefficient = 118.7, 95% confidence interval [CI] = 87.6 to 149.8) and the chewing duration (unstandardized regression coefficient = 1.7, 95% CI = 1.3 to 2.2). These associations remained significant after adjusting for sociodemographic status, health behaviors, and health status (multivariable models 1 and 2 in Table 3). We obtained consistent associations after further adjusting for the number of natural teeth (multivariable model 3 in Table 3). The unstandardized regression coefficients (95% CI) were 115.6 (84.3 to 146.9) for the number of chews and 1.67 (1.2 to 2.1) for the chewing duration. Even after adjusting for masticatory performance or occlusal force, instead of the number of natural teeth, m-DVS remained positively associated with the number of chews and chewing duration (multivariable models 4 and 5 in Table 3). However, the m-DVS was not significantly associated with the chewing speed (unstandardized regression coefficient = 0.26, 95% CI = −0.18 to 0.70, Multivariable model 3 in Table 3). The results of models 1 through 5, including unstandardized regression coefficients, 95% CI, and *p*-values for covariates, are listed in Table S1. The results of subgroup analysis by sex are shown in Tables S2 and S3. The results were similar to those in the primary analysis.

## 4. Discussion

This study investigated the association between dietary variety, assessed using photographs, and masticatory behaviors, measured with wearable devices among community-dwelling older adults. The m-DVS was significantly and positively associated with the number of chews and longer chewing duration. Furthermore, these associations remained significant even after adjusting for oral health and function. To the best of our knowledge, this study is the first to identify the association between consuming a variety of foods and masticatory behaviors in the daily diet among community-dwelling older adults.

We observed that a higher m-DVS was associated with a greater number of chews and longer chewing duration. We propose the following explanation for this association: consuming a wide variety of foods may increase the frequency of meals that include staple foods, main dishes, and side dishes, which is a traditional Japanese dietary pattern [4]. People who frequently consume meals consisting of staple foods, main dishes, and side dishes tend to chew their food slowly and thoroughly [33]. Compared to single-dish meals, such as a rice bowl, a meal with staple foods, main dishes, and side dishes generally involves more plates [34]. Additionally, meals that include a staple food, a main dish, and a side dish generally contain more vegetables [35]. Since vegetables are high in dietary fiber, they likely require more chews and longer chewing duration to form a swallowable food bolus compared to other food groups. As the frequency of meal patterns that combine staple meals, main dishes, and side dishes increases, the number of side dishes consisting mainly of vegetables also increases [36], which may lead to an increase in the number of chews and chewing duration for the meal as a whole. Based on this possibility, future research should examine whether increased consumption of side dishes and vegetables, along with a great number of dishes, correlates with chewing frequency.

However, no significant association was found between m-DVS and chewing speed. One previous study reported that when chewing speed was intentionally varied, no correlation with mastication efficiency was found [37]. This suggests that chewing speed follows an individualized rhythm that is less susceptible to external influences. Chewing speed is thought to develop over time, particularly in older adults. Unlike the number of chews and chewing duration, which can be influenced by food hardness and texture [18,38], chewing speed is likely a characteristic strongly influenced by individual traits.

The present study has various strengths. First, the masticatory behaviors in daily meals were objectively measured using an ear-worn bite sensor. Second, the variety of food groups in meals was objectively evaluated by registered dietitians based on photographs. Therefore, they were not subject to recall bias, highlighting the value of the objective in our research. Nevertheless, our study has some limitations. First, the data collection period was short (at least three days). Whereas the DVS is typically assessed by asking participants to recall what they have eaten for the past week, this study used the m-DVS, which used at least three days of dietary records. The time period was shortened to reduce the burden of data collection on participants, which would help to prevent dropouts. As a result, none of the participants dropped out after the initiation of at-home measurements. A previous study [4] indicated that a correlation likely exists between m-DVS and DVS. In addition, a checklist of dietary variety per meal has already been used in local communities [25,26]. Although we did not examine the validity and reliability of the m-DVS, the accuracy of the m-DVS scores was assessed by trained registered dietitians. Furthermore, this short duration may not represent the daily eating habits of the participants. Therefore, it remains unclear whether increasing the variety of foods in daily meals contributes to an increase in the number of chews. Further studies should extend the measurement period and evaluate the association between the DVS and m-DVS. Nevertheless, as only usual meals were measured, these data are considered to reflect daily eating habits. Second, certain bias may have occurred among the participants in this study, as they were required not only to wear BS but also to operate a smartphone. Consequently, recruitment conditions included requirements related to smartphone and computer use. A study including individuals who do not use the Internet might yield different results regarding the association between dietary variety and masticatory behaviors. However, we believe that the potential individual differences in smartphone and BS operation during data collection were minimized owing to the recruitment conditions for devices use and the provision of sufficient explanations beforehand. Third, because we used self-administered questionnaires, items regarding educational status, number of household members, perceived financial situation, and CCI might have created some bias in the data. Fourth, there might have also been selection bias. In addition to ear-worn bite sensors, this study required participants to use a smartphone. In Japan, the proportion of older adults who use the internet is 70% for those aged 70–79 years and 30% for those aged 80 years and over [39]. As a result, the exclusion rate for this study was 61.4%, which is very high, because those unable to exchange messages via smartphones or computers were not eligible to participate. A previous study reported that individuals who used the Internet were more likely to engage in healthy behaviors [40]. Therefore, a further study that includes non-Internet users may yield different results on the association between dietary variety and masticatory behaviors. Future studies should encourage participation irrespective of smartphone usage to enhance the generalizability of the findings.

## 5. Conclusions

Our study demonstrates that consuming a greater variety of foods was associated with a higher number of chews and a longer chewing duration among community-dwelling older adults in Japan. Consuming a variety of foods may contribute to thorough chewing.

## Figures and Tables

**Table 1:** Participant characteristics (n = 63).

	Mean (SD)	n (Percentage)
Basic information		
Sex, female		27 (42.9%)
Age, years	74.4 (3.1)	
Educational status (years of schooling)	13.9 (2.3)	
Number of household members		
One person		12 (19.0%)
Two people		37 (58.7%)
Three or more people		14 (22.2%)
Perceived financial situation		
Very comfortable		2 (3.2%)
Comfortable		28 (44.4%)
Average		32 (50.8%)
Difficult		1 (1.6%)
Very difficult		0 (0%)
BMI, kg/m^2^	23.1 (3.3)	
CCI, score	0.5 (0.9)	
Oral health and function		
Number of natural teeth	21.4 (7.3)	
Masticatory performance, score	4.7 (2.2)	
Occlusal force, N	504.0 (199.5)	

BMI, body mass index; CCI, Charlson Comorbidity Index; SD, standard deviation.

**Table 2:** Characteristics of mealtime data (n = 587).

Masticatory Behaviors	Mean (SD)	n (Percentage)
The number of chews, cycles	1211.5 (723.6)	
The chewing duration, minutes	16.4 (9.8)	
The chewing speed, cycle/minutes	73.1 (12.2)	
Dietary variety		
The m-DVS, number of food groups	4.4 (1.6)	
Food groups included in one meal		
Meat		328 (55.9%)
Fish and shellfish		230 (39.2%)
Eggs		210 (35.8%)
Soybean products		207 (35.3%)
Milk and dairy products		197 (33.6%)
Green and yellow vegetables		447 (76.1%)
Seaweed		209 (35.6%)
Fruits		205 (34.9%)
Potatoes		94 (16.0%)
Oils and fats		457 (77.9%)

m-DVS, modified Dietary Variety Score; SD, standard deviation.

**Table 3:** Association between dietary variety and masticatory behaviors (n = 587).

		The Number of Chews (Cycles)			The Chewing Duration (Minutes)			The Chewing Speed (Cycle/Min)		
	Exposure Variable	b	95% CI	*p*-Value	b	95% CI	*p*-Value	b	95% CI	*p*-Value
Univariable model										
	m-DVS (per 1 increase)	118.7	87.6 to 149.8	<0.01	1.7	1.3 to 2.2	<0.01	0.3	−0.2 to 0.7	0.21
Multivariable model										
Model 1 *	m-DVS (per 1 increase)	119.5	88.4 to 150.6	<0.01	1.7	1.3 to 2.2	<0.01	0.3	−0.2 to 0.7	0.21
Model 2 ^†^	m-DVS (per 1 increase)	116.4	85.1 to 147.7	<0.01	1.7	1.3 to 2.1	<0.01	0.3	−0.2 to 0.7	0.25
Model 3 ^‡^	m-DVS (per 1 increase)	116.5	85.2 to 147.8	<0.01	1.7	1.3 to 2.2	<0.01	0.3	−0.2 to 0.7	0.26
Model 4 ^§^	m-DVS (per 1 increase)	115.9	84.5 to 147.2	<0.01	1.7	1.3 to 2.1	<0.01	0.2	−0.2 to 0.7	0.28
Model 5 ^¶^	m-DVS (per 1 increase)	116.0	84.7 to 147.3	<0.01	1.7	1.3 to 2.1	<0.01	0.3	−0.2 to 0.7	0.25

* Adjusted for sex and age. ^†^ Adjusted for covariates from model 1 and further adjusted for years of education, number of household members, perceived financial situation, body mass index, and Charlson Comorbidity Index. ^‡^ Adjusted for covariates from model 2 and further adjusted for number of natural teeth. ^§^ Adjusted for covariates from model 2 and further adjusted for masticatory performance. ^¶^ Adjusted for covariates from model 2 and further adjusted for occlusal force. m-DVS, modify Dietary Variety Score; b, unstandardized regression coefficient; CI, confidence interval.

## Data Availability

The data presented in this study are accessible upon request from the corresponding author. However, the data are not publicly available due to the ethical and legal restrictions enforced by the Ethics Committee of the Tokyo Metropolitan Institute for Geriatrics and Gerontology.

## Supplementary Materials

The following supporting information can be downloaded at https://www.mdpi.com/article/10.3390/nu17040695/s1: Table S1: Multivariate models for the association between dietary variety and masticatory behaviors; Table S2: Multivariate models for the association between dietary variety and masticatory behaviors of male participants; Table S3: Multivariate models for the association between dietary variety and masticatory behaviors of female participants.
