# Supplementary material for: Association Between Dietary Variety and Masticatory Behaviors Measured Using Wearable Device Among Community-Dwelling Older Adults in Japan: A Multilevel Meal-by-Meal Analysis
Source: Nutrients. 2025 Feb 15;17(4):695. doi: 10.3390/nu17040695 (PMC11858430; doi:10.3390/nu17040695)
Supplement: Supplementary file 1 [file nutrients-17-00695-s001.zip › Supplementary Table S1_nutrients-3415615.pdf]

Supplementary information

Table S1. Multivariate models for the association between dietary variety and masticatory behaviors (n=587)

		The number of chews (Cycles)			The chewing duration (Minutes)			The chewing speed (Cycle/Min)		
	Exposure variables	b	95% CI	P-value	b	95% CI	P-value	b	95% CI	P-value
Multivariable model										
Model 1*	The m-DVS (per 1 increase)	119.5	88.4 to 150.6	<0.01	1.7	1.3 to 2.2	<0.01	0.3	-0.2 to 0.7	0.21
	Sex	78.0	-165.3 to 321.3	0.53	0.5	-2.6 to 3.7	0.74	1.4	-3.7 to 6.5	0.59
	Age	39.5	0.6 to 78.4	0.05	0.5	-0.04 to 1.0	0.07	0.3	0.5 to 1.2	0.41
Model 2†	The m-DVS (per 1 increase)	116.4	85.1 to 147.7	<0.01	1.7	1.3 to 2.1	<0.01	0.3	-0.2 to 0.7	0.25
	Sex	82.0	-213.1 to 377.2	0.59	1.0	-2.7 to 4.7	0.59	0.7	-5.5 to 6.9	0.82
	Age	34.6	-6.0 to 75.1	0.09	0.4	-0.1 to 0.9	0.15	0.2	-0.6 to 1.1	0.62
	Years of education	30.7	-28.7 to 90.1	0.31	0.6	-0.2 to 1.3	0.13	-0.3	-1.6 to 0.9	0.59
	Number of household members									
	One person	Ref.								
	Two people	-259.0	-588.4 to 70.4	0.12	-4.6	-8.7 to -0.5	0.03	3.4	-3.5 to 10.4	0.33
	Three or more people	-373.2	-764.5 to 18.0	0.06	-5.9	-10.8 to -1.0	0.02	1.9	-6.4 to 10.1	0.66
	Perceived financial situation									
	Difficult	Ref.								
	Average	554.5	-427.8 to 1536.7	0.27	5.6	-6.7 to 17.8	0.37	25.1	4.3 to 45.9	0.02
	Comfortable	610.9	-378.0 to 1599.8	0.23	5.8	-6.6 to 18.1	0.36	26.6	5.6 to 47.5	0.01
	Very comfortable	1016.5	-178.6 to 2211.7	0.10	9.9	-5.1 to 24.8	0.20	29.9	4.6 to 55.2	0.02
	BMI	-20.7	-61.9 to 20.5	0.32	-0.1	-0.7 to 0.4	0.57	-0.2	-1.1 to 0.6	0.61
	CCI	51.2	-86.4 to 188.7	0.47	0.9	-0.9 to 2.6	0.32	-1.5	-4.4 to 1.4	0.31

Model 3‡	The m-DVS (per 1 increase)	116.5	85.2 to 147.8	<0.01	1.7	1.3 to 2.2	<0.01	0.3	-0.2 to 0.7	0.26
	Sex	67.9	-246.7 to 382.4	0.67	0.8	-3.2 to 4.7	0.70	1.6	-5.0 to 8.2	0.63
	Age	33.6	-7.8 to 75.1	0.11	0.4	-0.2 to 0.9	0.18	0.3	-0.6 to 1.1	0.54
	Years of education	31.0	-29.0 to 91.0	0.31	0.6	-0.2 to 1.3	0.13	-0.4	-1.6 to 0.9	0.57
	Number of household members									
	One person	Ref.								
	Two people	-252.3	-588.2 to 83.6	0.14	-4.5	-8.7 to -0.3	0.04	3.0	-4.0 to 10.0	0.40
	Three or more people	-366.8	-764.4 to 30.7	0.07	-5.8	-10.8 to -0.8	0.02	1.5	-6.9 to 9.8	0.73
	Perceived financial situation									
	Difficult	Ref.								
	Average	544.8	-448.9 to 1538.6	0.28	5.4	-7.0 to 17.8	0.39	25.7	4.8 to 46.6	0.02
	Comfortable	607.3	-391.1 to 1605.7	0.23	5.7	-6.8 to 18.2	0.37	26.8	5.8 to 47.8	0.01
	Very comfortable	967.4	-289.2 to 2224.1	0.13	9.0	-6.7 to 24.8	0.26	33.1	6.6 to 59.5	0.01
	BMI	-22.3	-65.4 to 20.8	0.31	-0.2	-0.7 to 0.4	0.52	-0.1	-1.0 to 0.8	0.78
	CCI	50.1	-88.9 to 189.2	0.48	0.9	-0.9 to 2.6	0.34	-1.4	-4.4 to 1.5	0.34
	Number of natural teeth	-2.8	-23.1 to 17.4	0.78	-0.05	-0.3 to 0.2	0.72	0.2	-0.2 to 0.6	0.41
Model 4§	The m-DVS (per 1 increase)	115.9	84.5 to 147.2	<0.01	1.7	1.3 to 2.1	<0.01	0.2	-0.2 to 0.7	0.28
	Sex	113.6	-196.2 to 423.4	0.47	1.3	-2.6 to 5.1	0.53	2.6	-3.8 to 8.9	0.43
	Age	36.1	-4.9 to 77.2	0.08	0.4	-0.1 to 0.9	0.14	0.3	-0.5 to 1.1	0.48
	Years of education	26.0	-35.1 to 87.2	0.40	0.5	-0.2 to 1.3	0.17	-0.6	-1.9 to 0.6	0.33
	Number of household members									
	One person	Ref.								

	Two people	-278.7	-614.3 to 57.0	0.10	-4.7	-9.0 to -0.5	0.03	2.3	-4.6 to 9.2	0.51
	Three or more people	-376.5	-769.7 to 16.7	0.06	-5.9	-10.9 to -1.0	0.02	1.7	-6.3 to 9.7	0.68
	Perceived financial situation									
	Difficult	Ref.								
	Average	569.3	-418.6 to 1557.2	0.26	5.7	-6.7 to 18.1	0.37	25.9	5.7 to 46.2	0.01
	Comfortable	617.4	-376.5 to 1611.2	0.22	5.8	-6.7 to 18.3	0.36	26.9	6.5 to 47.3	0.01
	Very comfortable	1138.5	-111.2 to 2388.1	0.07	10.8	-4.9 to 26.5	0.18	37.0	11.4 to 62.6	<0.01
	BMI	-19.4	-61.0 to 22.2	0.36	-0.1	-0.7 to 0.4	0.60	-0.1	-1.0 to 0.7	0.73
	CCI	54.4	-84.1 to 192.9	0.44	0.9	-0.8 to 2.6	0.31	-1.3	-4.2 to 1.5	0.36
	Masticatory performance	23.3	-42.8 to 89.5	0.49	0.2	-0.7 to 1.0	0.67	1.3	-0.01 to 2.7	0.05
Model 5¶	The m-DVS (per 1 increase)	116.0	84.7 to 147.3	<0.01	1.7	1.3 to 2.1	<0.01	0.3	-0.2 to 0.7	0.25
	Sex	118.8	-205.9 to 443.6	0.47	1.6	-2.4 to 5.7	0.43	0.6	-6.3 to 7.5	0.86
	Age	33.2	-8.0 to 74.3	0.11	0.3	-0.2 to 0.9	0.18	0.2	-0.7 to 1.1	0.63
	Years of education	27.6	-33.2 to 88.4	0.37	0.5	-0.2 to 1.3	0.18	-0.3	-1.6 to 0.9	0.60
	Number of household members									
	One person	Ref.								
	Two people	-278.1	-616.6 to 60.4	0.11	-4.9	-9.1 to -0.7	0.02	3.5	-3.7 to 10.6	0.34
	Three or more people	-388.2	-785.7 to 9.2	0.06	-6.2	-11.1 to -1.2	0.02	1.9	-6.5 to 10.3	0.66
	Perceived financial situation									
	Difficult	Ref.								
	Average	585.5	-409.7 to 1580.6	0.25	6.1	-6.4 to 18.5	0.34	25.0	3.9 to 46.2	0.02
	Comfortable	646.0	-357.5 to 1649.5	0.21	6.4	-6.2 to 18.9	0.32	26.5	5.2 to 47.8	0.01
	Very comfortable	1133.6	-139.7 to 2406.9	0.08	11.8	-4.1 to 27.7	0.15	29.7	2.7 to 56.7	0.03

BMI	-17.8	-60.6 to 25.0	0.41	-0.1	-0.6 to 0.4	0.71	-0.2	-1.1 to 0.7	0.61
CCI	54.5	-84.5 to 193.5	0.44	0.9	-0.8 to 2.7	0.29	-1.5	-4.5 to 1.4	0.32
Occlusal force	0.2	-0.5 to 1.0	0.58	0.004	-0.01 to 0.01	0.46	-0.0005	-0.02 to 0.02	0.95

*Adjusted for sex and age.

†Adjusted for covariates from model 1 and further adjusted for years of education, number of household members, perceived financial situation, body mass index, and Charlson Comorbidity Index.

‡Adjusted for covariates from model 2 and further adjusted for number of natural teeth.

§Adjusted for covariates from model 2 and further adjusted for masticatory performance.

¶Adjusted for covariates from model 2 and further adjusted for occlusal force.

m-DVS, modify Dietary Variety Score; BMI, body mass index; CCI, Charlson Comorbidity Index; b, unstandardized regression coefficient; CI, confidence interval
