# Supplementary material for: Association Between Dietary Variety and Masticatory Behaviors Measured Using Wearable Device Among Community-Dwelling Older Adults in Japan: A Multilevel Meal-by-Meal Analysis
Source: Nutrients. 2025 Feb 15;17(4):695. doi: 10.3390/nu17040695 (PMC11858430; doi:10.3390/nu17040695)
Supplement: Supplementary file 1 [file nutrients-17-00695-s001.zip › Supplementary Table S2_nutrients-3415615.pdf]

Supplementary information

Table S2. Multivariate models for the association between dietary variety and masticatory behaviors of male participants (n=339)

		The number of chews (Cycles)			The chewing duration (Minutes)			The chewing speed (Cycle/Min)		
	Exposure variables	b	95% CI	P-value	b	95% CI	P-value	b	95% CI	P-value
Univariable model										
	The m-DVS (per 1 increase)	140.8	96.3 to 185.2	<0.01	2.1	1.4 to 2.7	<0.01	0.3	-0.3 to 0.9	0.26
Multivariable model										
Model 1*	The m-DVS (per 1 increase)	141.7	97.2 to 186.2	<0.01	2.1	1.5 to 2.7	<0.01	0.3	-0.3 to 0.9	0.26
	Age	32.0	-15.8 to 79.8	0.19	0.4	-0.2 to 1.1	0.18	-0.03	-1.1 to 1.0	0.95
Model 2†	The m-DVS (per 1 increase)	138.3	93.3 to 183.3	<0.01	2.1	1.4 to 2.7	<0.01	0.3	-0.3 to 0.9	0.31
	Age	26.7	-26.7 to 80.2	0.33	0.4	-0.3 to 1.1	0.32	-0.3	-1.3 to 0.8	0.64
	Years of education	37.6	-37.3 to 112.4	0.32	0.7	-0.3 to 1.7	0.18	-0.2	-1.7 to 1.3	0.82
	Number of household members									
	One person	Ref.								
	Two people	-117.7	-576.7 to 341.3	0.61	-2.4	-8.4 to 3.6	0.44	3.1	-6.2 to 12.4	0.51
	Three or more people	-172.5	-782.5 to 437.5	0.58	-2.7	-10.7 to 5.4	0.51	1.6	-10.8 to 14.0	0.80
	Perceived financial situation									
	Difficult	Ref.								
	Average	609.0	-417.3 to 1635.3	0.24	6.4	-7.1 to 19.9	0.35	25.2	4.4 to 46.1	0.02
	Comfortable	625.7	-410.9 to 1662.2	0.24	5.1	-8.5 to 18.7	0.46	29.6	8.5 to 50.7	0.01
	Very comfortable	971.6	-267.2 to 2210.5	0.12	8.7	-7.6 to 25.0	0.29	31.2	6.1 to 56.4	0.02
	BMI	-18.9	-73.4 to 35.5	0.49	-0.2	-0.9 to 0.5	0.64	0.1	-1.0 to 1.2	0.86

	CCI	35.3	-160.3 to 230.8	0.72	0.7	-1.9 to 3.3	0.59	-2.1	-6.1 to 1.9	0.31
	The m-DVS (per 1 increase)	138.6	93.8 to 183.4	<0.01	2.1	1.4 to 2.7	<0.01	0.3	-0.3 to 0.9	0.30
	Age	-1.8	-55.4 to 51.9	0.95	-0.01	-0.7 to 0.7	0.99	-0.3	-1.5 to 0.9	0.62
	Years of education	48.8	-20.0 to 117.7	0.16	0.8	-0.1 to 1.7	0.08	-0.2	-1.7 to 1.4	0.84
	Number of household members									
	One person	Ref.								
	Two people	94.9	-355.8 to 545.6	0.68	0.3	-5.7 to 6.3	0.92	3.5	-6.7 to 13.7	0.50
	Three or more people	59.8	-526.1 to 645.7	0.84	0.3	-7.5 to 8.1	0.94	1.9	-11.3 to 15.2	0.77
Model 3‡	Perceived financial situation									
	Difficult	Ref.								
	Average	608.2	-327.6 to 1543.9	0.20	6.4	-6.0 to 18.8	0.31	25.2	3.9 to 46.5	0.02
	Comfortable	559.2	-387.4 to 1505.7	0.25	4.3	-8.2 to 16.9	0.50	29.5	8.0 to 51.0	0.01
	Very comfortable	177.1	-1114.7 to 1468.8	0.79	-1.3	-18.5 to 15.8	0.88	29.9	0.6 to 59.2	0.05
	BMI	-24.3	-74.2 to 25.5	0.34	-0.2	-0.9 to 0.4	0.48	0.1	-1.0 to 1.2	0.87
	CCI	-32.3	-218.1 to 153.5	0.73	-0.1	-2.6 to 2.3	0.91	-2.2	-6.4 to 2.1	0.31
	Number of natural teeth	-47.3	-84.5 to -10.0	0.01	-0.6	-1.1 to -0.1	0.02	-0.1	-0.9 to 0.8	0.85
	The m-DVS (per 1 increase)	137.9	92.7 to 183.1	<0.01	2.1	1.4 to 2.7	<0.01	0.3	-0.3 to 0.9	0.33
	Age	27.5	-27.3 to 82.2	0.32	0.4	-0.4 to 1.1	0.33	-0.2	-1.3 to 0.9	0.71
	Years of education	34.9	-44.1 to 114.0	0.39	0.7	-0.4 to 1.7	0.21	-0.4	-1.9 to 1.2	0.66
Model 4§	Number of household members									
	One person	Ref.								
	Two people	-141.3	-646.0 to 363.4	0.58	-2.4	-9.1 to 4.2	0.48	1.5	-8.6 to 11.6	0.77

	Three or more people	-173.3	-794.8 to 448.1	0.58	-2.7	-10.8 to 5.5	0.52	1.5	-10.9 to 14.0	0.81
	Perceived financial situation									
	Difficult	Ref.								
	Average	604.8	-441.4 to 1651.0	0.26	6.4	-7.4 to 20.1	0.36	25.0	4.0 to 46.0	0.02
	Comfortable	627.5	-428.7 to 1683.8	0.24	5.1	-8.7 to 19.0	0.47	29.7	8.5 to 50.9	0.01
	Very comfortable	1042.5	-345.8 to 2430.8	0.14	8.7	-9.5 to 27.0	0.35	36.1	8.3 to 64.0	0.01
	BMI	-20.6	-77.7 to 36.5	0.50	-0.2	-0.9 to 0.6	0.66	-0.01	-1.2 to 1.1	0.99
	CCI	46.5	-172.3 to 265.2	0.68	0.7	-2.2 to 3.6	0.62	-1.3	-5.7 to 3.1	0.56
	Masticatory performance	15.0	-107.7 to 137.8	0.81	0.01	-1.6 to 1.6	0.99	1.0	-1.4 to 3.5	0.40
Model 5¶	The m-DVS (per 1 increase)	138.9	93.8 to 184.0	<0.01	2.1	1.4 to 2.7	<0.01	0.3	-0.3 to 0.9	0.30
	Age	28.7	-26.3 to 83.8	0.31	0.4	-0.3 to 1.1	0.30	-0.2	-1.4 to 0.9	0.67
	Years of education	41.1	-36.6 to 118.8	0.30	0.7	-0.3 to 1.7	0.17	-0.1	-1.7 to 1.4	0.86
	Number of household members									
	One person	Ref.								
	Two people	-91.9	-572.3 to 388.5	0.71	-2.1	-8.4 to 4.2	0.52	3.4	-6.4 to 13.1	0.50
	Three or more people	-141.4	-776.3 to 493.4	0.66	-2.3	-10.6 to 6.1	0.59	1.8	-11.1 to 14.7	0.78
	Perceived financial situation									
	Difficult	Ref.								
	Average	574.7	-479.5 to 1628.9	0.28	6.0	-7.9 to 19.8	0.40	25.0	3.4 to 46.5	0.02
	Comfortable	577.4	-497.7 to 1652.5	0.29	4.5	-9.6 to 18.6	0.53	29.2	7.3 to 51.2	0.01
	Very comfortable	812.0	-627.3 to 2251.3	0.27	6.7	-12.2 to 25.6	0.49	29.9	0.6 to 59.2	0.05

BMI	-20.7	-76.5 to 35.2	0.47	-0.2	-0.9 to 0.5	0.61	0.10	-1.0 to 1.2	0.88
CCI	23.7	-181.7 to 229.1	0.82	0.6	-2.1 to 3.3	0.68	-2.2	-6.4 to 2.0	0.31
Occlusal force	-0.3	-1.5 to 1.0	0.65	-0.004	-0.02 to 0.01	0.66	-0.002	-0.03 to 0.02	0.86

*Adjusted for age.

†Adjusted for covariates from model 1 and further adjusted for years of education, number of household members, perceived financial situation, body mass index, and Charlson Comorbidity Index.

‡Adjusted for covariates from model 2 and further adjusted for number of natural teeth.

§Adjusted for covariates from model 2 and further adjusted for masticatory performance.

¶Adjusted for covariates from model 2 and further adjusted for occlusal force.

m-DVS, modify Dietary Variety Score; BMI, body mass index; CCI, Charlson Comorbidity Index; b, unstandardized regression coefficient; CI, confidence interval
