# Supplementary material for: Association Between Dietary Variety and Masticatory Behaviors Measured Using Wearable Device Among Community-Dwelling Older Adults in Japan: A Multilevel Meal-by-Meal Analysis
Source: Nutrients. 2025 Feb 15;17(4):695. doi: 10.3390/nu17040695 (PMC11858430; doi:10.3390/nu17040695)
Supplement: Supplementary file 1 [file nutrients-17-00695-s001.zip › Supplementary Table S3_nutrients-3415615.pdf]

***Supplementary information***

**Table S3.** Multivariate models for the association between dietary variety and masticatory behaviors of female participants (n=248)

| | | The number of chews (Cycles) | | | The chewing duration (Minutes) | | | The chewing speed (Cycle/Min) | | |
|---|---|---|---|---|---|---|---|---|---|---|
| | Exposure variables | b | 95% CI | P-value | b | 95% CI | P-value | b | 95% CI | P-value |
| Univariable model | | | | | | | | | | |
| | The m-DVS (per 1 increase) | 78.7 | 38.5 to 118.8 | <0.01 | 1.1 | 0.6 to 1.6 | <0.01 | 0.2 | -0.5 to 0.8 | 0.62 |
| Multivariable model | | | | | | | | | | |
| Model 1* | The m-DVS (per 1 increase) | 79.0 | 38.9 to 119.1 | <0.01 | 1.1 | 0.6 to 1.6 | <0.01 | 0.2 | -0.5 to 0.8 | 0.61 |
| | Age | 52.4 | -15.8 to 120.6 | 0.13 | 0.5 | -0.3 to 1.4 | 0.24 | 0.9 | -0.4 to 2.2 | 0.16 |
| Model 2† | The m-DVS (per 1 increase) | 76.8 | 36.4 to 117.1 | <0.01 | 1.1 | 0.5 to 1.6 | <0.01 | 0.1 | -0.5 to 0.8 | 0.69 |
| | Age | 38.9 | -48.4 to 126.2 | 0.38 | 0.3 | -0.7 to 1.3 | 0.56 | 0.8 | -1.0 to 2.5 | 0.39 |
| | Years of education | 12.2 | -138.5 to 162.9 | 0.87 | 0.3 | -1.4 to 2.1 | 0.70 | -1.4 | -4.4 to 1.7 | 0.37 |
| | Number of household members | | | | | | | | | |
| | One person | Ref. | | | | | | | | |
| | Two people | -447.3 | -1088.4 to 193.8 | 0.17 | -6.9 | -14.4 to 0.5 | 0.07 | 3.4 | -9.5 to 16.3 | 0.60 |
| | Three or more people | -589.0 | -1284.6 to 106.6 | 0.10 | -9.2 | -17.3 to -1.1 | 0.03 | 1.8 | -12.2 to 15.9 | 0.80 |
| | Perceived financial situation | | | | | | | | | |
| | Average | Ref. | | | | | | | | |
| | Comfortable | 34.9 | -425.4 to 495.3 | 0.88 | 1.0 | -4.4 to 6.3 | 0.72 | -2.1 | -11.4 to 7.1 | 0.65 |
| | BMI | -28.2 | -111.6 to 55.2 | 0.51 | -0.2 | -1.1 to 0.8 | 0.75 | -1.1 | -2.8 to 0.6 | 0.20 |
| | CCI | 58.2 | -180.5 to 296.8 | 0.63 | 0.8 | -1.9 to 3.6 | 0.56 | -0.3 | -5.1 to 4.5 | 0.90 |
| Model 3‡ | The m-DVS (per 1 increase) | 76.2 | 35.8 to 116.6 | <0.01 | 1.1 | 0.5 to 1.6 | <0.01 | 0.1 | -0.5 to 0.8 | 0.71 |

| | | | | | | | | | | |
|---|---|---|---|---|---|---|---|---|---|---|
| | Age | 35.0 | -53.0 to 122.9 | 0.43 | 0.3 | -0.8 to 1.3 | 0.62 | 0.7 | -1.1 to 2.5 | 0.44 |
| | Years of education | 23.9 | -129.2 to 177.0 | 0.76 | 0.5 | -1.3 to 2.3 | 0.60 | -1.2 | -4.3 to 1.9 | 0.45 |
| | Number of household members | | | | | | | | | |
| | One person | Ref. | | | | | | | | |
| | Two people | -532.2 | -1199.1 to 134.7 | 0.12 | -7.9 | -15.6 to -0.1 | 0.05 | 2.0 | -11.6 to 15.5 | 0.77 |
| | Three or more people | -647.1 | -1355.2 to 61.1 | 0.07 | -9.9 | -18.1 to -1.6 | 0.02 | 0.9 | -13.5 to 15.2 | 0.91 |
| | Perceived financial situation | | | | | | | | | |
| | Average | Ref. | | | | | | | | |
| | Comfortable | -56.2 | -555.5 to 443.0 | 0.82 | -0.02 | -5.8 to 5.8 | 1.0 | -3.7 | -13.8 to 6.5 | 0.48 |
| | BMI | -8.1 | -101.7 to 85.4 | 0.86 | 0.1 | -1.0 to 1.2 | 0.91 | -0.8 | -2.7 to 1.1 | 0.43 |
| | CCI | 36.4 | -207.2 to 280.1 | 0.77 | 0.6 | -2.2 to 3.4 | 0.68 | -0.7 | -5.6 to 4.3 | 0.79 |
| | Number of natural teeth | 15.1 | -16.3 to 46.5 | 0.34 | 0.2 | -0.2 to 0.5 | 0.38 | 0.3 | -0.4 to 0.9 | 0.43 |
| Model 4§ | The m-DVS (per 1 increase) | 76.4 | 36.0 to 116.8 | <0.01 | 1.1 | 0.5 to 1.6 | <0.01 | 0.1 | -0.5 to 0.8 | 0.69 |
| | Age | 38.8 | -50.0 to 127.6 | 0.39 | 0.3 | -0.7 to 1.3 | 0.57 | 0.8 | -0.9 to 2.4 | 0.37 |
| | Years of education | 7.6 | -146.4 to 161.7 | 0.92 | 0.3 | -1.5 to 2.1 | 0.74 | -1.6 | -4.5 to 1.3 | 0.27 |
| | Number of household members | | | | | | | | | |
| | One person | Ref. | | | | | | | | |
| | Two people | -488.6 | -1154.3 to 177.2 | 0.15 | -7.3 | -15.1 to 0.5 | 0.07 | 1.2 | -11.4 to 13.8 | 0.85 |
| | Three or more people | -610.0 | -1320.8 to 100.8 | 0.09 | -9.4 | -17.7 to -1.1 | 0.03 | 0.7 | -12.7 to 14.1 | 0.92 |
| | Perceived financial situation | | | | | | | | | |
| | Average | Ref. | | | | | | | | |
| | Comfortable | 4.7 | -473.8 to 483.2 | 0.98 | 0.7 | -4.9 to 6.3 | 0.80 | -3.8 | -12.8 to 5.2 | 0.41 |

| | | | | | | | | | | |
|---|---|---|---|---|---|---|---|---|---|---|
| | BMI | -18.9 | -109.0 to 71.2 | 0.68 | -0.1 | -1.1 to 1.0 | 0.88 | -0.6 | -2.3 to 1.1 | 0.49 |
| | CCI | 42.0 | -206.3 to 290.4 | 0.74 | 0.7 | -2.2 to 3.6 | 0.64 | -1.2 | -5.8 to 3.5 | 0.63 |
| | Masticatory performance | 33.4 | -74.8 to 141.6 | 0.54 | 0.3 | -1.0 to 1.6 | 0.65 | 1.8 | -0.2 to 3.8 | 0.08 |
| Model 5¶ | The m-DVS (per 1 increase) | 76.1 | 35.7 to 116.5 | <0.01 | 1.1 | 0.5 to 1.6 | <0.01 | 0.1 | -0.5 to 0.8 | 0.70 |
| | Age | 37.1 | -49.8 to 124.1 | 0.40 | 0.3 | -0.7 to 1.3 | 0.59 | 0.8 | -1.0 to 2.6 | 0.40 |
| | Years of education | 17.1 | -133.1 to 167.3 | 0.82 | 0.4 | -1.3 to 2.1 | 0.63 | -1.4 | -4.5 to 1.7 | 0.38 |
| | Number of household members | | | | | | | | | |
| | One person | Ref. | | | | | | | | |
| | Two people | -563.7 | -1234.6 to 107.2 | 0.10 | -8.6 | -16.3 to -1.0 | 0.03 | 3.5 | -10.5 to 17.4 | 0.63 |
| | Three or more people | -627.9 | -1323.3 to 67.6 | 0.08 | -9.8 | -17.7 to -1.9 | 0.02 | 1.8 | -12.6 to 16.3 | 0.80 |
| | Perceived financial situation | | | | | | | | | |
| | Average | Ref. | | | | | | | | |
| | Comfortable | 23.3 | -435.1 to 481.7 | 0.92 | 0.8 | -4.4 to 6.0 | 0.76 | -2.1 | -11.7 to 7.4 | 0.66 |
| | BMI | -8.7 | -98.7 to 81.4 | 0.85 | 0.1 | -0.90 to 1.15 | 0.81 | -1.1 | -3.0 to 0.8 | 0.25 |
| | CCI | 44.6 | -194.0 to 283.2 | 0.71 | 0.6 | -2.1 to 3.4 | 0.65 | -0.3 | -5.3 to 4.7 | 0.91 |
| | Occlusal force | 0.7 | -0.5 to 1.8 | 0.27 | 0.01 | -0.004 to 0.023 | 0.16 | -0.0002 | -0.02 to 0.02 | 0.99 |

*Adjusted for age.

†Adjusted for covariates from model 1 and further adjusted for years of education, number of household members, perceived financial situation, body mass index, and Charlson Comorbidity Index.

‡Adjusted for covariates from model 2 and further adjusted for number of natural teeth.

§Adjusted for covariates from model 2 and further adjusted for masticatory performance.

¶Adjusted for covariates from model 2 and further adjusted for occlusal force.

m-DVS, modify Dietary Variety Score; BMI, body mass index; CCI, Charlson Comorbidity Index; b, unstandardized regression coefficient; CI, confidence interval
